# Shifts in Fecal Metabolite Profiles Associated With Ramadan Fasting Among Chinese and Pakistani Individuals

**DOI:** 10.3389/fnut.2022.845086

**Published:** 2022-05-03

**Authors:** Siyu Chen, Ikram Ali, Xin Li, Danfeng Long, Ying Zhang, Ruijun Long, Xiaodan Huang

**Affiliations:** ^1^School of Public Health, Lanzhou University, Lanzhou, China; ^2^College of Ecology, Lanzhou University, Lanzhou, China

**Keywords:** fasting, metabolite, gut microbiota, diet, ethnic groups

## Abstract

The human gut microbiota has been proposed to serve as a multifunctional organ in host metabolism, contributing effects to nutrient acquisition, immune response, and digestive health. Fasting during Ramadan may alter the composition of gut microbiota through changes in dietary behavior, which ultimately affects the contents of various metabolites in the gut. Here, we used liquid chromatography–mass spectrometry-based metabolomics to investigate the composition of fecal metabolites in Chinese and Pakistani individuals before and after Ramadan fasting. Principal component analysis showed distinct separation of metabolite profiles among ethnic groups as well as between pre- and post-fasting samples. After Ramadan fasting, the Chinese and Pakistani groups showed significant differences in their respective contents of various fecal metabolites. In particular, L-histidine, lycofawcine, and cordycepin concentrations were higher after Ramadan fasting in the Chinese group, while brucine was enriched in the Pakistani group. The KEGG analysis suggested that metabolites related to purine metabolism, 2-oxocarboxylic acid metabolism, and lysine degradation were significantly enriched in the total subject population pre-fasting vs. post-fasting comparisons. Several bacterial taxa were significantly correlated with specific metabolites unique to each ethnic group, suggesting that changes in fecal metabolite profiles related to Ramadan fasting may be influenced by associated shifts in gut microbiota. The fasting-related differences in fecal metabolite profile, together with these group-specific correlations between taxa and metabolites, support our previous findings that ethnic differences in dietary composition also drive variation in gut microbial composition and diversity. This landscape view of interconnected dietary behaviors, microbiota, and metabolites contributes to the future development of personalized, diet-based therapeutic strategies for gut-related disorders.

## Introduction

Ramadan fasting is a religious rite of Muslims which is also recognized as a form of intermittent fasting. More than 1.5 billion Muslims reportedly refrain from eating or drinking from sunrise (Sahur) to sunset (Iftar) during the holy month of Ramadan each year, which lasts between 28 and 30 days (1, 2), and thus practice intermittent fasting and caloric restriction. Intermittent fasting has been shown to have various beneficial health effects, including improved immune system function (3, 4), enhanced cognitive function (5), improved body composition and reduced obesity (6, 7), and even reduced episodes of seizures in some patients with epilepsy (8). However, the mechanisms mediating the effects of intermittent fasting remain largely obscure, which hamper the adoption of intermittent fasting as a strategy for improving health or as a disease intervention. Experiments in mice and clinical observations suggest that the benefits of intermittent fasting, such as positive effects on body weight and metabolism, can be explained by reduced energy intake (9). In addition, intermittent fasting regimens can influence metabolic regulation through changes in modifiable lifestyle behaviors, circadian rhythms, and gut microbiota (10). Our previous study has revealed that Ramadan fasting can significantly influence gut microbiota through dietary changes and has highlighted the enrichment of specific bacterial taxa, such as *Sutterella* and *Parabacteroides*, with implied health benefits in some individuals (11).

Gut microbiota have been described as a new metabolic organ that participates in regulating host metabolism (12, 13). Accumulating evidence suggests that changes in metabolic levels can affect health status and are correlated with some diseases, such as Type 2 diabetes (14), pulmonary tuberculosis (15), obesity (16), epilepsy (17), and others. Recent studies have reported that both environmental and host factors, such as environmental contaminants (18), lifestyle (19), disease conditions (20), and drug use (21), could all influence gut metabolism. To explore the role of caloric restriction-induced microbiome changes in ischemic stroke rehabilitation, Huang et al. (22) applied metabolite profiling in a mouse model. Their results showed that caloric restriction increased the levels of several metabolites, such as prostatin B1 and 3β-hydroxy-5-cholic acid, and that the upregulation of these metabolites was correlated with the abundance of bifidobacteria, suggesting that caloric restriction leads to enrichment for both specific gut microbiota and their associated metabolites. Previous studies (23, 24) have also reported that intermittent fasting can confer protective effects against diabetes by impacting gut microbiota, which consequently modulates the levels of circulating microbial metabolites. Intermittent fasting can also regulate the composition of intestinal microbial communities in diabetic patients, ultimately affecting the levels of circulating microbial metabolites. Wang et al. (25) also proposed that the beneficial effects of caloric restriction on health and metabolism might be attributable to shifts in the composition and diversity of gut microbiota. However, changes in gut metabolism driven by Ramadan fasting, with consideration for host- or ethnicity-related factors, remain poorly characterized, indicating that a detailed analysis of correlations among fasting, ethnicity, dietary composition, fecal metabolites, and gut microbiota is warranted. In light of our previous data that showed diet-related differences in gut microbiota are affected by Ramadan fasting in Pakistani and Chinese groups living in close proximity, we proposed that the month-long Ramadan fasting could affect gut metabolite profiles through the response by gut microbiota to intermittent fasting.

In this study, we investigated the influence of fasting during Ramadan on the gut metabolic profiles in fecal samples obtained from healthy Chinese and Pakistani subjects living in Lanzhou, China, using liquid chromatography–mass spectrometry (LC-MS)-based metabolomics analysis. This study was conducted with three main objectives. First, we sought to better understand whether caloric restriction during Ramadan fasting is a determining factor for metabolite profiles. Second, this study aimed to identify differences in metabolite profiles between the Pakistani and Chinese Hui ethnic groups, and to investigate whether these differences were diet-related. The third objective of this work was to test whether the diversity of intestinal flora is correlated with metabolite profile.

## Materials and Methods

### Ethical Approval, Consent to Participate, and Recruitment

All procedures performed were approved by the Medical Ethics Committee of the School of Public Health at Lanzhou University (GW-20171013). Participants received the detailed information about the study and informed consent before sampling.

Participants' mental and physical health were included as criteria. We excluded volunteers in case of (1) gastrointestinal diseases, chronic diseases, anorexia nervosa, and cachexia, (2) insufficiency of liver and kidney, (3) smoking or drinking alcohol, (4) Bristol Stool Form Scale (<3/>4), and (5) antibiotics usage within last 3 months (26). According to the inclusion and exclusion criteria, 34 healthy adults (aged 18–40 years) living in a proximity area in Lanzhou city (16 Chinese adults and 18 Pakistani adults) were recruited. All participants signed the consent letter and attended Ramadan fasting from 15 May 2018 to 15 June 2018; both dietary survey (3 days 24-h food dietary recall) and biosamples (fecal) were collected before and after Ramadan fasting (27). To figure out the gut metabolic profile driven by fasting, all the participants were subject to three groups, namely, (1) total fasting group: total before fasting (TBF) vs. total after fasting (TAF); (2) Pakistani fasting group: Pakistani before fasting (PBF) vs. Pakistani after fasting (PAF); and (3) Chinese fasting group: Chinese before fasting (CBF) vs. Chinese after fasting (CAF). In contrast, group comparisons between ethnic groups were carried out by CBF vs. PBF and CAF vs. PAF.

### Sample Collection and 16S rRNA Gene Sequencing

Fecal samples were collected for all participants on the morning of 15 May 2018 (before fasting) and 15 June 2018 (after fasting). Samples were frozen in liquid nitrogen immediately upon receipt to maintain sample stability, then transferred to the laboratory, and further stored at −70°C for future experiments.

As described by Ali et al. (11), QIAamp DNA Stool Mini Kit (QIAGEN, Hilden, Germany) was used for DNA extraction and subjected to DNA sequencing using MiSeq Reagent Kit version 3 (Illumina, San Diego, CA, USA). Raw reads were loaded into the European Nucleotide Archive under the succession number PRJEB38231 (http://www.ebi.ac.uk/ena/data/view/PRJEB38231), and the results have been presented in previous studies (11).

### LC-MS Experiments

The samples were slowly thawed on the ice. A 50-mg sample was added into a 1.5-ml centrifuge tube, followed by adding 800 μl of 80% methanol, grinding for 90 s at 65 Hz, thorough mixing using eddy oscillation, and treating using ultrasound at 4°C for 30 min. The mixture was kept at −40°C for 1 h, followed by mixing using eddy oscillation for 30 s at 4°C and allowing to stand for 0.5 h. All supernatants were placed into a centrifuge tube, allowed to stand at −40°C for 1 h, and centrifuged at 4°C and 12,000 rpm for 15 min. A 200 μl of supernatant was removed and 5 μl of internal standard (0.14 mg/ml dichlorophenylalanine) was added prior to transfer into injection vial. The treated samples were used for untargeted metabolomics analysis of metabolic characteristics, assisted by Shanghai Tianhao Biotechnology Co., Ltd.

The ACQUITY UPLC system (Thermo, Q Exactive) was used to analyze metabolic profiles in Electrospray ionization (ESI)-positive and ESI-negative modes. ACQUITY UPLC HSS T3 (2.1 × 100 mm, 1.8 μm) column was used for positive and negative modes. The binary gradient elution system consists of (A) water (containing 0.05% formic acid, V/V) and (B) acetonitrile, separated using the following gradient: 0 min, 5%B; 1 min, 5%B; 12 min, 95%B; 13.5 min, 95%B; 13.6 min, 5%B; and 16 min, 5%B. The flow rate was 0.3 ml/min, and the column temperature was 40°C.

Quality control (QC) samples that were included in batches of analytical samples during the course of the study were considered to monitor the overall quality of the sample pretreatment and mass spectrometry analyses. The data were collected using feature extraction and preprocessed using the Compound Discoverer software (Thermo), and then normalized and edited into two-dimensional data matrix using the Excel 2010 software, including retention time (RT), compound molecular weight (compMW), observations (samples), and peak intensity.

### Data Analysis

Unsupervised principal component analysis (PCA) was used to observe the overall distribution among samples and the degree of dispersion between groups. Then, supervised orthogonal partial least squares discrimination analysis (OPLS-DA) was used to distinguish the overall differences of metabolic profiles among groups and to search for the metabolites that differed between groups. In the OPLS-DA analysis, the Variable Importance for the Projection (VIP) > 1 was set as the difference variable. *T*-test combined with multivariate analysis OPLS-DA was used to screen out the metabolites that differed between groups (VIP > 1 and *p* < 0.05). Differential metabolites obtained from the comparison between groups were input into MetaboAnalyst 3.0. Cluster analysis was performed on different substances in different groups to check the relative changes in the contents of these different substances in different groups, and pathway attribution was carried out to locate the differential metabolites into metabolic pathways. Metabolite data were entered into the KEGG database using comprehensive analysis (e.g., enrichment analysis and topological analysis) of the pathways where the differential metabolites were located, and we could further screen the pathways and find the key pathways with the highest correlation based on the difference in metabolites.

The Wilcoxon signed-rank tests were used to compare the “before vs. after Ramadan fasting” groups (CBF vs. CAF, PBF vs. PAF, TBF vs. TAF), and the Mann–Whitney *U* test was used for comparisons between ethnic groups (CBF vs. PBF, CAF vs. PAF). In addition, Pearson's correlation coefficient was used to analyze the correlation between fecal metabolite and the relative abundance of intestinal microorganisms.

The SPSS version 23.0 was used for all statistical analyses. R (phearmap) was used to analyze the correlation between fecal metabolites and intestinal flora, and Pearson's correlation coefficient (the Spearman statistical analysis) was used to indicate the degree of correlation (28). The correlation coefficient is presented in the form of heat map, and the correlation is reflected by a color gradient.

## Results

### Demographic and Dietary Intake Features of Study Participants

The dietary intake and demographic characteristics of all participants in this study were previously described by Ali et al. (11). A total of 34 volunteers (16 Chinese and 18 Pakistanis) were included in this study, aged between 20 and 33 years, with a male-to-female ratio of approximately 8:2. Body mass index (BMI) of subjects before and after fasting was calculated based on the height and weight, with significantly greater BMI in the Chinese group before fasting (*p* = 0.01) but no significant difference between groups after fasting (Supplementary Table 1). Before fasting, the Chinese group had higher daily intake of grains (CBF range = 537.35–921.35 g vs. PBF range = 487.33–666.68 g) and soybeans (CBF range = 27.91–226.67 g vs. PBF = 0.00 g), but a lower daily intake of livestock meat (CBF range = 10.11–87.83 g vs. PBF range = 41.67–127.09 g), fruits (CBF range = 0.00–82.92 g vs. PBF range = 0.00–251.10 g), and poultry (CBF = 0.00 g vs. PBF range = 79.16–244.99 g), compared to that consumed by the Pakistani group. The proportions of energy sources consumed by each group showed that carbohydrate intake was significantly greater among Chinese participants (CBF range = 61.50–70.18% vs. PBF range = 45.24–54.73%), while the Pakistani group consumed a significantly greater proportion of fats (PBF range = 31.86–38.23% vs. CBF range = 16.71–25.61%) and proteins (PBF = 13.15–17.81% vs. CBF = 11.41–14.49%). No significant differences were identified between groups in beverage consumption, including caffeinated and non-caffeinated beverages (11).

### Shifts in Fecal Metabolite Profiles Associated With Fasting or Ethnicity

To identify the differences in fecal metabolite profiles attributable to either Ramadan fasting or ethnicity, we used untargeted LC-MS for metabolomics analysis of fecal supernatants from before and after Ramadan fasting. Metabolite profiles were then subjected to PCA modeling to identify differences between ethnic groups (CBF vs. PBF and CAF vs. PAF) or within ethnic groups before and after Ramadan fasting (CBF vs. CAF and PBF vs. PAF), and among total subjects before and after fasting (TBF vs. TAF). A total of 257 different metabolites were detected between before and after samples from total subjects (VIP > 1, *p* < 0.05). In contrast, 118 metabolites were different after fasting in the Chinese groups (CBF vs. CAF) and 500 metabolites were unique to pre- or post-fasting Pakistani groups (PBF vs. PAF) (VIP > 1, *p* < 0.05, Supplementary Figure 2). Furthermore, 547 metabolites differed between Chinese and Pakistani ethnic groups before fasting (CBF vs. PBF), while 796 metabolites differed between ethnic groups after fasting (CAF vs. PAF) (VIP > 1, *p* < 0.05, Supplementary Figure 2).

#### Fasting Groups

**Figure 1:**
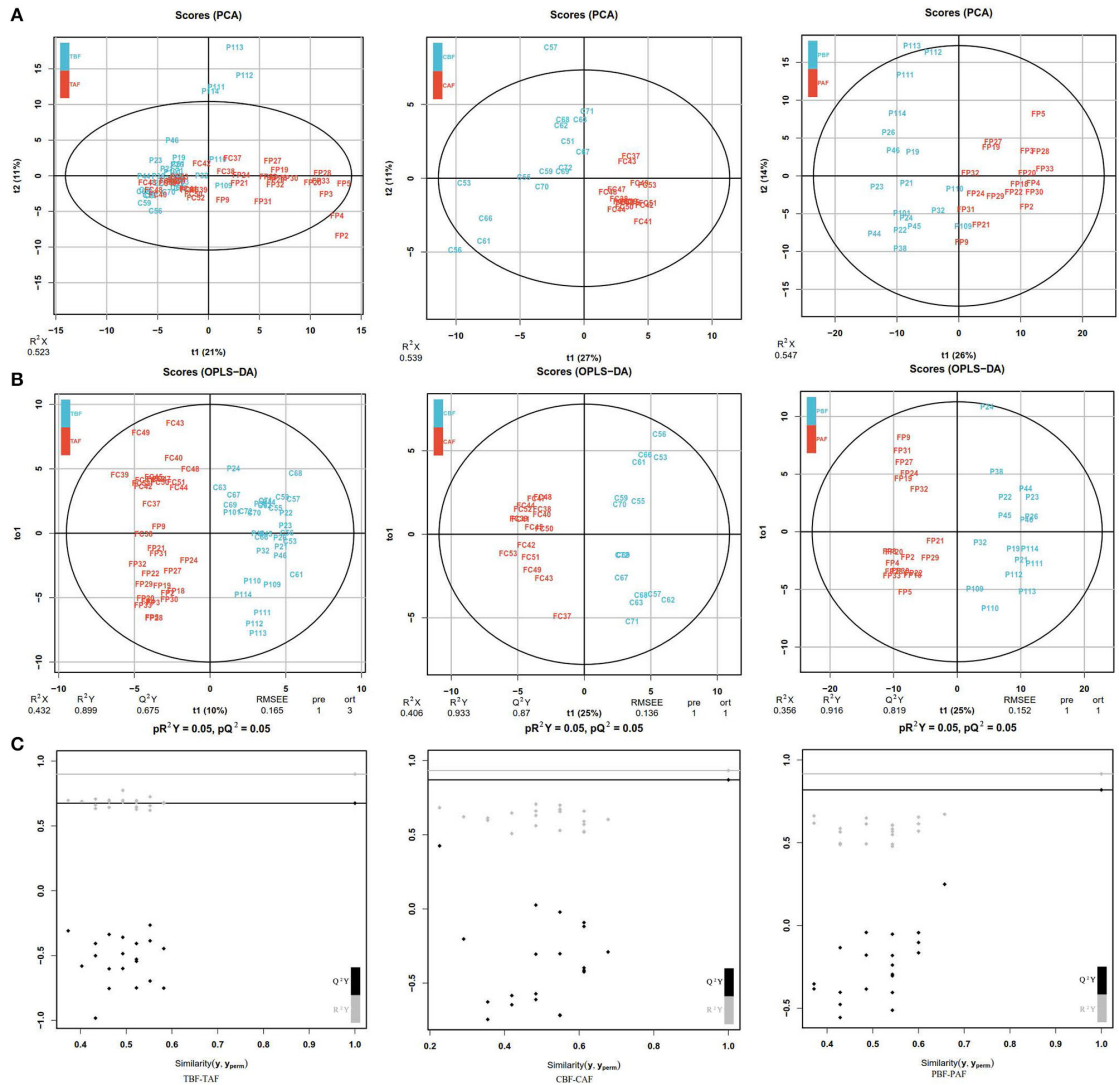
**(A)** Principal component analysis (PCA) analyses comparing metabolic profiles of subjects within each ethnicity group before and after fasting (CBF vs. CAF, PBF vs. PAF) and among the total study subjects (TBF vs. TAF). Each numbered datapoint represents an individual subject. Blue, before fasting; Red, after fasting; CBF/CAF, Chinese before or after fasting; PBF/PAF, Pakistani before or after fasting; TBF/TAF, Total subjects before or after fasting. **(B)** Orthogonal partial least squares discrimination analysis (OPLS-DA) scores of fasting groups showed significant differences in clustering between samples collected before and after fasting (TBF vs. TAF; CBF vs. CAF; PBF vs. PAF). The abscissa represents the predicted principal component score of the first principal component and the ordinate represents the variability within the grouping. R^2^ represents the explanatory power of the model to variables, and Q^2^ represents the predictability of the model. **(C)** Model validation of OPLS-DA (TBF vs. TAF; CBF vs. CAF; PBF vs. PAF) (*p* < 0.05). The abscissa indicates similarity with the original model, and the ordinates represent R^2^Y and Q values. Gray and black points on the respective lines in the upper right corner indicate the actual values, while points on the left represent simulated values. When all simulated Q^2^ (black) and R^2^ values (gray) are lower than the respective original points, the model is robust, without over-fitting.

The PCA analysis showed significant differences between pre- and post-fasting metabolite profiles for both ethnic groups (CBF vs. CAF and PBF vs. PAF) and in the total subject population (TBF vs. TAF) (Figure 1A). Total subjects clustered into distinct before and after fasting groups along PC1 (21%)/PC2 (11%) axes, with some overlap in the groups. In comparison with each ethnic group, the pre- and post-fasting samples also formed distinct clusters that were largely explained by the PC1(27%)/PC2(11%) in CBF vs. CAF comparisons and PC1(26%)/PC2(14%) in PBF vs. PAF comparisons (Figure 1A). Modeling by OPLS-DA (Figure 1B) showed more obvious separation in metabolite profiles before and after Ramadan fasting for both ethnic groups, which was supported by model validation (Figure 1C) for each of these OPLS-DA plots. These results showed that, despite ethnic differences, fecal metabolite contents and composition were changed after fasting.

#### Ethnic Groups

We next identified the differences in fecal metabolite profiles before and after fasting that were attributable to ethnicity. PCA showed that the significant separation of metabolite profiles between Chinese and Pakistani groups before fasting (CBF vs. PBF) could be largely explained by PC1 (31%)/PC2 (8%), as were differences in metabolite profiles between ethnic groups after fasting PC1 (32%)/PC2 (9%) (CAF vs. PAF). Moreover, the separation was more obvious after fasting (Supplementary Figure 1A). OPLS-DA modeling (Supplementary Figure 1B) further revealed significant differences in the clustering of samples, indicating that fecal metabolite profiles were distinct to each ethnic group, both before (CBF vs. PBF) and after (CAF vs. PAF) fasting, which was supported by model validation (Supplementary Figure 1C). Taken together, these results showed that fecal metabolites were distinct to each ethnic group, and that these differences persisted after fasting.

### Differential Enrichment for Metabolites and KEGG Pathways After Fasting

**Figure 2:**
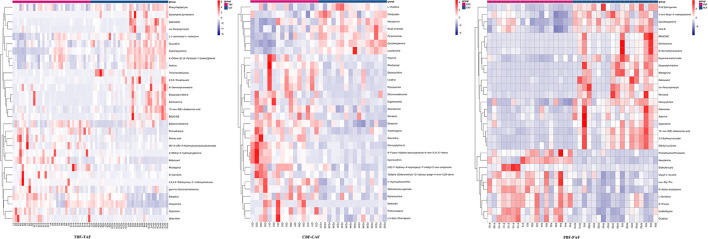
Heat maps of metabolites of fasting groups (TBF vs. TAF; CBF vs. CAF; PBF vs. PAF) show the first 30 metabolite that was significantly different before and after fasting (*p* < 0.05).

**Figure 3:**
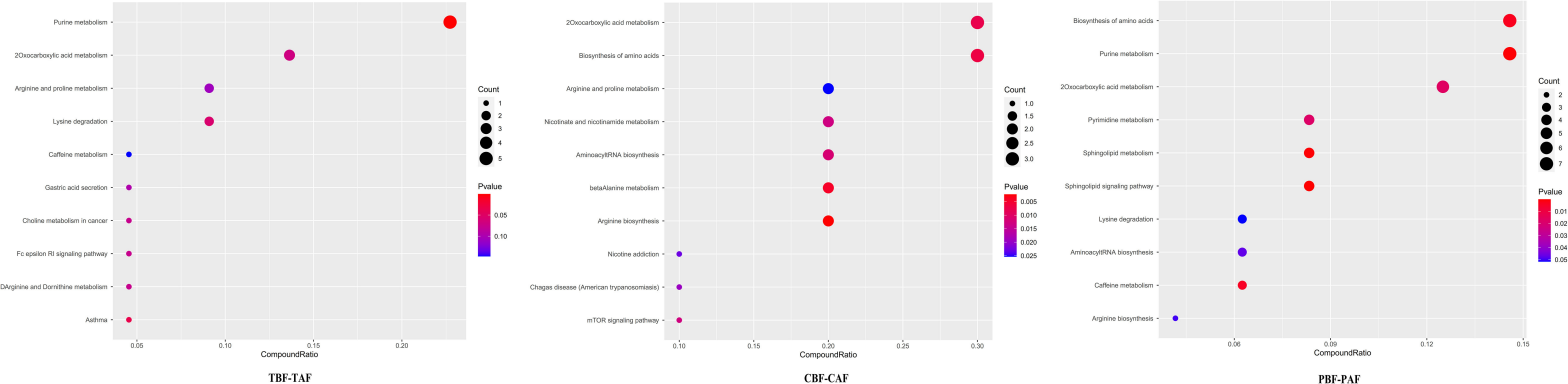
Dot plot of top 10 metabolite pathway of fasting groups (TBF vs. TAF; CBF vs. CAF; PBF vs. PAF. TBF/TAF, Total subjects before or after fasting; CBF/CAF, Chinese before or after fasting; PBF/PAF, Pakistani before or after fasting). The abscissa is the proportion of metabolites, and the ordinate is the path. The redder the color of the point, the smaller the *p*-value, and the size of the point represents the quantity of metabolites.

To explore the potential mechanisms responsible for changes in fecal metabolites before and after fasting, we analyzed the significantly different metabolites and their respective metabolic pathways. Heat map visualization of differentially abundant metabolites between pre- and post-fasting groups showed that the levels of ethephon, zearalenone, trachelogenin, deoxycytidine, and rhodopinal were higher before fasting than after among Chinese participants, while L-histidine, cordycepin, hexylamine, pyrazinamide, and cyclohexylamine were increased after fasting. Among Pakistani subjects, metabolites, such as promethazine, diethofencarb, hexylamine, D-proline, and *N*-α-acetyl-lysine, were all higher before fasting, whereas cyclohexylamine, virol B, ecgonine methyl ester, deoxycytidine, and adenine levels were all significantly higher after fasting compared to their pre-fasting levels (Figure 2). The KEGG analysis suggested that purine metabolism, 2-oxocarboxylic acid metabolism, and lysine degradation were all significantly enriched among the total subject population in pre- and post-fasting comparisons (TBF vs. TAF). In contrast, 2-oxocarboxylic acid metabolism, biosynthesis of amino acids, and arginine biosynthesis pathways were significantly enriched in comparisons of metabolites before and after fasting specifically among Chinese subjects (CBF vs. CAF). Among Pakistani participants, pathways related to biosynthesis of amino acids, purine metabolism, and 2-oxocarboxylic acid metabolism were significantly enriched in before and after fasting comparisons (PBF vs. PAF) (Figure 3).

Comparisons of significantly different metabolites between ethnic groups before fasting (CBF vs. PBF) indicated that ethephon, diethofencarb, sodium oleate, montanol, and nafcillin were enriched in samples from Chinese subjects, while picolinic acid, rehmaionoside C, homobaldrinal, cyclohexylamine, and N6-trimethyl-L-lysine were all higher in Pakistani subjects. After fasting (CAF vs. PAF), diethofencarb, soyasapogenol E, norcodeine, ethephon, and hexylamine were higher in the Chinese group, while ergothioneine, dicrotophos, nabilone, bufadienolide, and luffariellolide were enriched in the Pakistani group (Supplementary Figure 3). Kyoto Encyclopedia of Genes and Genomes (KEGG) pathway analysis showed that biosynthesis of amino acids, caffeine metabolism, arginine, and proline metabolism were significantly enriched in comparison with pre-fasting metabolites between ethnic groups (CBF vs. PBF), while pathways related to biosynthesis of amino acids, arginine and proline metabolism, and ABC transporters were significantly enriched after fasting (CAF vs. PAF) (Supplementary Figure 4).

### Fecal Metabolites Show an Obvious Correlation With Gut Microbiota Before and After Fasting

**Figure 4:**
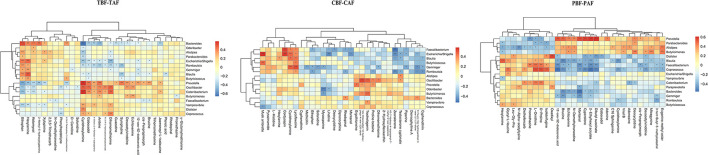
Correlation between fecal microbiota members and metabolites between fasting groups (TBF vs. TAF; CBF vs. CAF; PBF vs. PAF. TBF/TAF, Total subjects before or after fasting; CBF/CAF, Chinese before or after fasting; PBF/PAF, Pakistani before or after fasting). The abscissa represents the top 30 differential metabolites, and the ordinate represents the highest abundance species of intestinal microflora (genus level). Black stars in the box indicate significant results (*p < 0.05; **p < 0.01).

We then evaluated possible correlations between fecal metabolites and intestinal microbiota between ethnic groups before and after fasting using Pearson's correlation analysis. Correlations between the top 30 most abundant metabolites and gut microbiota (genus level) in different groups (1. TBF vs. TAF; 2. CBF vs. CAF; 3. PBF vs. PAF; 4. CBF vs. PBF; 5. CAF vs. PAF) are shown in Figure 4 and Supplementary Figure 5.

Among the total subjects (TBF vs. TAF), *Prevotella* was positively correlated with cyclohexylamine (cor ≥ 0.5, *p* < 0.01) but negatively correlated with ethephon (cor ≤ −0.5, *p* < 0.01), whereas *Bacteroides* was positively correlated with ethephon (cor ≥ 0.5, *p* < 0.01) and negatively correlated with cyclohexylamine (cor ≤ −0.5, *p* < 0.01). *Catenibacterium* was positively correlated with cyclohexylamine (cor ≥ 0.5, *p* < 0.01). In the pre- and post-fasting Chinese groups (CBF and CAF), *Escherichia/Shigella* was positively correlated with musk ambrette and cordycepin (cor ≥ 0.5, *p* < 0.01), while *Faecalibacterium* was positively correlated with cordycepin (cor ≥ 0.5, *p* < 0.01) and negatively correlated with zearalenone (cor ≤ −0.5, *p* < 0.01). *Gemmiger* showed a positive correlation with lycofawcine (cor ≥ 0.5, *p* < 0.01), and *Oscillibacter* was positively correlated with 12α-chloromethyl-12-hydroxy-pregn-4-ene-3, 20-dione (cor ≥ 0.5, *p* < 0.01).

Comparisons of Pakistan groups before and after fasting (PBF and PAF) revealed a positive correlation between *Prevotella* and 12-oxo-9Z-dodecenoic acid, 2-ethylhexyl acrylate, montanol, and cyperolone (cor ≥ 0.5, *p* < 0.01). *Coprococcus* was positively correlated with L-ornithine, D-proline, and Isofebrifugine (cor ≥ 0.5, *p* < 0.01), but was negatively correlated with 12-oxo-9Z-dodecenoic acid, 2-ethylhexyl acrylate, dibutyl succinate, and cyperolone (cor ≤ −0.5, *p* < 0.01). *Blautia* abundance was positively correlated with hexylamine (cor ≥ 0.5, *p* < 0.01) and negatively correlated with brucine (cor ≤ −0.5, *p* < 0.01), while *Dialister* was positively correlated with oxadixyl (cor ≥ 0.5, *p* < 0.01), and *Bacteroides* was negatively correlated with montanol (cor ≤ −0.5, *p* < 0.01) (Figure 4).

Comparisons of pre-fasting ethnic groups (CBF vs. PBF) showed a negative correlation between *Prevotella* and nafcillin, 9-fluoro-16α-hydroxyandrost-4-ene-3,11,17-trione, and cyphenothrin (cor ≤ −0.7, *p* < 0.01). *Oscillibacter* was negatively correlated with trans-trans-farnesyl phosphate and sodium oleate (cor ≤ −0.7, *p* < 0.01), and *Faecalibacterium* was negatively correlated with ethephon (cor ≤ −0.7, *p* < 0.01). *Catenibacterium* shared a positive relationship with azacitidine (cor ≥ 0.7, *p* < 0.01), but was negatively correlated with 17α-methyl-5α-androstane-3β-11β-17β-triol and trans-trans-farnesyl phosphate (cor ≤ −0.7, *p* <0.01). *Bacteroides* was positively correlated with 9-fluoro-16α-hydroxyandrost-4-ene-3-11-17-trione (cor ≥ 0.7, *p* < 0.01) and negatively correlated with leonuridine, cyclohexylamine, theobromine, N6-trimethyl-L-lysine, cis-acetylacrylate, picolinic acid, gaboxadol, calystegin A3, gabaculine, methionine sulfoxide, and carteolol (cor ≤ −0.7, *p* < 0.01).

Correlation analysis after fasting (CAF vs. PAF) showed that romboutsia was negatively correlated with N6-trimethyl-L-lysine and calystegin A3 (cor ≤ −0.7, *p* < 0.01). *Prevotella* was positively correlated with N6-trimethyl-L-lysine, methylone, calystegin A3, dicyclomine, kikkanol B, and deoxycytidine (cor ≥ 0.7, *p* < 0.01). *Oscillibacter* had positive correlations with homobaldrinal, cyclohexylamine, nabilone, methylone, cis-acetylacrylate-2-hydroxy-2,4-pentadienoate, dicrotophos, dioctyl phthalate, cassaidine, bufadienolide, and kikkanol B (cor ≥ 0.7, *p* < 0.01), but was negatively correlated with norcodeine (cor ≤ −0.7, *p* < 0.01). *Escherichia/Shigella* was negatively correlated with N6-trimethyl-L-lysine, rehmaionoside C, and testosterone enanthate (cor ≤ −0.7, *p* < 0.01), while *Bacteroides* was negatively correlated with 6-eenzylaminopurine (cor ≤ −0.7, *p* < 0.01) (Supplementary Figure 5).

## Discussion

To date, several studies have examined the effects of Ramadan fasting on gut microbiota. However, the results have been inconsistent, largely due to heterogeneity in age, sex, ethnicity, and/or health status, as well as in methodology. In addition, the effects of dietary behavior (e.g., Ramadan fasting) on the relationship between gut microbiota and fecal metabolites have not yet been investigated. In this study, we applied untargeted metabolomics to detect differential metabolites between Chinese and Pakistani ethnic groups before and after fasting for Ramadan to identify characteristic fecal metabolites and enriched KEGG pathways that are potentially influenced by either dietary behavior (fasting) or host factors (ethnicity), or both.

In previous study, we investigated whether Ramadan fasting leads to changes in gut microbiota composition in 34 healthy participants from China or Pakistan by conducting high-throughput 16S rRNA gene sequencing of fecal samples before and after fasting. We detected a total of 1,074 operational taxonomic unit (OTUs) in the full study population and identified several taxa as indicators of host ethnicity and/or changes in dietary behavior. Notably, *Dorea, Klebsiella*, and *Faecalibacterium* were more abundant after fasting in the Chinese group, while *Sutterella, Parabacteroides*, and *Alistipes* were significantly increased after fasting in the Pakistani group. Further analysis of gut microbiota and dietary composition by principal co-ordinates analysis (PCoA) showed similar patterns of slight separation of samples across fasting groups, but dramatic distinctions between ethnic groups. These findings, together with obvious correlations between dietary intake and microbial composition, strongly suggested that differences in gut microbiota among the two ethnic groups were likely driven by differences in dietary intake (11).

Intermittent fasting has been shown to trigger substantial remodeling of the gut microbiota (29). For example, Ozkul et al. reported significant enrichment of *Lachnospiraceae* and *Erysipelotrichaceae* after fasting, which was associated with the accumulation of short-chain fatty acids (30). However, our previous study has showed enrichment for butyric acid-producing bacteria, such as *Clostridium_XlVa* and *Lachnospiraceae incertae sedis*, before Ramadan fasting rather than after. We are inclined to speculate that regional differences in the sources of food consumed during and after Ramadan fasting (i.e., different food sources than those in other published studies) led to a decline in this population, rather than enrichment, but further evidence examining microbiota in food sources from geographically isolated regions is necessary to test this possibility.

In this work, fecal metabolomics profiles from before and after fasting in Chinese and Pakistani groups revealed that L-histidine, lycofawcine, and cordycepin, among others, were significantly higher after fasting in Chinese participants. L-histidine is reported to provide potentially ameliorative effects on atopic dermatitis and was shown to exhibit some neuroprotective activity, notably in the treatment of vascular dementia (31, 32). Intestinal dysfunction is commonly reported in the studies on calorie restriction, especially intermittent fasting (33). However, the accumulation of functional metabolites produced by gut bacteria with anti-inflammatory effects, such as propionic acid, has been proposed to possibly mitigate intestinal dysfunction during intermittent fasting (34). Lycofawcine is a bioactive alkaloid obtained from *Lycopodium* that reportedly exerts anti-inflammatory and anti-cytotoxic biological activities (35), while cordycepin exerts significant protective effects against hepatic steatosis, inflammation, liver injury, and fibrosis in mice under metabolic stress through activation of the Adenosine 5'-monophosphate (AMP)-activated protein kinase (AMPK) signaling pathway (36).

Among the Pakistani participants, brucine content was significantly higher after fasting than before. Brucine is an anti-inflammatory and analgesic drug used to relieve arthritis and traumatic pain (37). It was also shown to inhibit tumor angiogenesis, growth, and bone metastasis by downregulating the expression of vascular endothelial growth factor (VEGF), and it can inhibit the growth and migration of Human colon cancer cell (LoVo) colorectal cancer cells by regulating the Wnt/β-catenin signaling pathway (38, 39). While Brucine increased, the alkaloid isofebrifugine decreased after fasting, which can significantly inhibit the proliferation, migration, and invasion of SGC7901 gastric cancer cells (40). It is unlikely that the observed enrichment for this compound was due to medical treatment and exclusion criteria for this study.

Metabolomics analysis in this study showed that diethofencarb, rehmaionoside C, soyasapogenol E, and bufadienolide levels, among other metabolites, differed between Chinese and Pakistani individuals. Moreover, the contents of caffeine and caffeine-specific metabolites, such as theobromine, paraxanthine, and 1,7-dimethyluric acid, were significantly higher in the fecal metabolites of Pakistani samples before fasting than in Chinese samples, which could reflect differences in dietary intake between the two groups (41), potentially reflecting differences in the consumption of chocolate, tea, soda, or coffee, the main dietary sources of caffeine (42).

Metabolites produced by a healthy gut microbiota, such as short-chain fatty acids (e.g., acetic, propionic, and butyric acids), play important roles in maintaining the intestinal barrier, (43) providing energy (44) and immune homeostasis (45). It is well-recognized that gut microbiota also participate in modulating the intestinal microenvironment and host metabolism, while the gut microecology is vulnerable to the effects of unhealthy diet (46) and antibiotic treatments (47). Furthermore, the disruption of gut microbial homeostasis may result in metabolic shifts, ultimately leading to the pathological development of metabolic diseases. Thus, identifying correlations between intestinal microbiota and fecal metabolites can enhance our understanding of intestinal microbiota function and regulatory effects on human health and disease. For example, a lifestyle intervention study and screen of gut microbiota with metabolite profiling by Jang and colleagues (48) showed that choline and betaine, which contribute to the risk of obesity, could serve as reliable biomarkers of metabolic changes driven by lifestyle interventions, and that their levels were affected by diet and gut microbiota. In our study, we observed a wide range of betaine levels after fasting, particularly in the Chinese group, but found no correlation between betaine and specific taxa, although other studies reported significant correlations between betaine levels and certain potentially beneficial bacteria. We thus speculated that this discrepancy may be related to the increased consumption of some betaine-rich foods during Ramadan, although further study is required to determine the dietary source of betaine in this group.

In summary, our findings suggest that intermittent fasting can affect the composition and diversity of intestinal microbiota, and hence microbial metabolites, possibly resulting in different effects on human health. Other studies have also shown that intermittent fasting can have profound beneficial effects on animal and human health (49–53), although fasting-based interventions most commonly focus on young, healthy participants and do not consider age- or disease-related differences in metabolism and other factors. For example, severe protein restriction results in weight loss in older, but not younger, mice; conversely, low protein intake is associated with reduced mortality in people aged 65 years and younger, but not in individuals aged 66 years and older (54). Currently, the majority of fasting-related studies suggest that this dietary behavior confers potentially beneficial effects on the human health. For example, in animal models, intermittent fasting can improve metabolic function and help control hormonal changes, inflammatory responses, lipid metabolism, and insulin sensitivity (55, 56). Although some studies show promising effects of intermittent fasting, the long-term effects of caloric restriction through this dietary method remain unclear (57). Some studies examining the effects of Ramadan fasting have shown that alertness is decreased in individuals during fasting (58, 59), while sleepiness and irritability are increased (60), or that fasting for Ramadan is associated with impairment of cognitive function (61). Thus, while most studies have shown that fasting is beneficial to human health, specific fasting strategies should be personalized based on an individual's current health status, dietary preferences, social environment, and other relevant factors.

## Conclusion

Our results suggest that fasting leads to changes in metabolite profiles specific to each ethnic group in a manner dependent on dietary components. These changes are correlated with dynamic shifts in microbiota composition and diversity which, in conjunction with dietary changes during Ramadan fasting, lead to enrichment or depletion of various functional metabolites. Future work should examine whether these changes in metabolite profiles are also correlated with the expression of biomarkers to determine if there are ethnicity-specific differences in adaptive response to Ramadan fasting. In addition, future work with more stringent controls will examine whether and to what extent environmental factors also contribute to shifts in metabolite profiles during Ramadan fasting.

## Data Availability Statement

The datasets presented in this study can be found in online repositories. The names of the repository/repositories and accession number(s) can be found in the article/Supplementary Material.

## Ethics Statement

The studies involving human participants were reviewed and approved by Medical Ethics Committee of School of Public Health in Lanzhou University. The patients/participants provided their written informed consent to participate in this study.

## Author Contributions

XH and RL: study design. XH: funding acquisition. SC and IA: investigation. SC, IA, XL, and DL: data analysis. SC: writing. XH, RL, and YZ: manuscript review and editing. All authors contributed to the article and approved the submitted version.

## Funding

This work was supported by research grants from NSFC (41875139, 82173497), the Fundamental Research Funds for the Central University (lzujbky – 2019-48) and the funds for International Cooperation Hub of Mountain Eco-Agriculture of Gansu Province.

## Conflict of Interest

The authors declare that the research was conducted in the absence of any commercial or financial relationships that could be construed as a potential conflict of interest.

## Publisher's Note

All claims expressed in this article are solely those of the authors and do not necessarily represent those of their affiliated organizations, or those of the publisher, the editors and the reviewers. Any product that may be evaluated in this article, or claim that may be made by its manufacturer, is not guaranteed or endorsed by the publisher.

## Supplementary Material

Supplementary Figure 1 **(A)** PCA analyses comparing metabolic profiles of subjects within Ethnic groups (CBF vs. PBF; CAF vs. PAF. CBF/PBF, Chinese or Pakistani before fasting; CAF/PAF, Chinese or Pakistani after fasting). Each numbered datapoint represents an individual subject. Blue, Pakistani; Red, Chinese. **(B)** OPLS-DA scores of fasting groups showed significant differences in clustering between ethnic groups (CBF vs. PBF; CAF vs. PAF). The abscissa represents the predicted principal component score of the first principal component and the ordinate represents the variability within the grouping. R^2^ represents the explanatory power of the model to variables, and Q^2^ represents the predictability of the model. **(C)** Model validation of OPLS-DA (CBF vs. PBF; CAF vs. PAF) (*p* < 0.05). The abscissa indicates similarity with the original model, and the ordinates represent R^2^Y and Q values. Gray and black points on the respective lines in the upper right corner indicate the actual values, while points on the left represent simulated values. When all simulated Q^2^ (black) and R^2^ values (gray) are lower than the respective original points, the model is robust, without over-fitting.

Supplementary Figure 2 **(A)** The volcano figure of fasting groups (TBF vs. TAF; CBF vs. CAF; PBF vs. PAF. CBF/CAF, Chinese before or after fasting; PBF/PAF, Pakistani before or after fasting; TBF/TAF, Total subjects before or after fasting) shows the number of different metabolites detected in different fasting groups (VIP > 1, *p* < 0.05), with red representing up-regulated metabolites and blue representing down-regulated metabolites. **(B)** The volcano figure of ethnic groups (CBF vs. PBF; CAF vs. PAF. CBF/PBF, Chinese or Pakistani before fasting; CAF/PAF, Chinese or Pakistani after fasting) shows the number of different metabolites detected in different ethnic groups (VIP > 1, *p* < 0.05), with red representing up-regulated metabolites and blue representing down-regulated metabolites.

Supplementary Figure 3 Heat maps of metabolites of ethnic groups (CBF vs. PBF; CAF vs. PAF. CBF/PBF, Chinese or Pakistani before fasting; CAF/PAF, Chinese or Pakistani after fasting) show the first 30 metabolite that was significantly different between Chinese and the Pakistanis (*p* < 0.05).

Supplementary Figure 4 Dot plot of top 10 metabolite pathway 619 of ethnic groups (CBF vs. PBF; CAF vs. PAF. CBF/PBF, Chinese or Pakistani before fasting; CAF/PAF, Chinese or Pakistani after fasting). The abscissa is the proportion of metabolites, and the ordinate is the path. The redder the color of the point, the smaller the *p* value, and the size of the point represents the quantity of metabolites.

Supplementary Figure 5 Correlation between fecal microbiota members and metabolites between of ethnic groups (CBF vs. PBF; CAF vs. PAF. CBF/PBF, Chinese or Pakistani before fasting; CAF/PAF, Chinese or Pakistani after fasting). The abscissa represents the top 30 differential metabolites, and the ordinate represents the highest abundance species of intestinal microflora (genus level). Black stars in the box indicate significant results (^*^*p* < 0.05; ^**^*p* < 0.01).

Supplementary Figure 6 Graphical Abstract: Bridging human health, gut microbiota & metabolites by fasting.
