# Supplementary figures and images for: Shifts in Fecal Metabolite Profiles Associated With Ramadan Fasting Among Chinese and Pakistani Individuals
Source: Front Nutr. 2022 May 3;9:845086. doi: 10.3389/fnut.2022.845086 (PMC9113920; doi:10.3389/fnut.2022.845086)

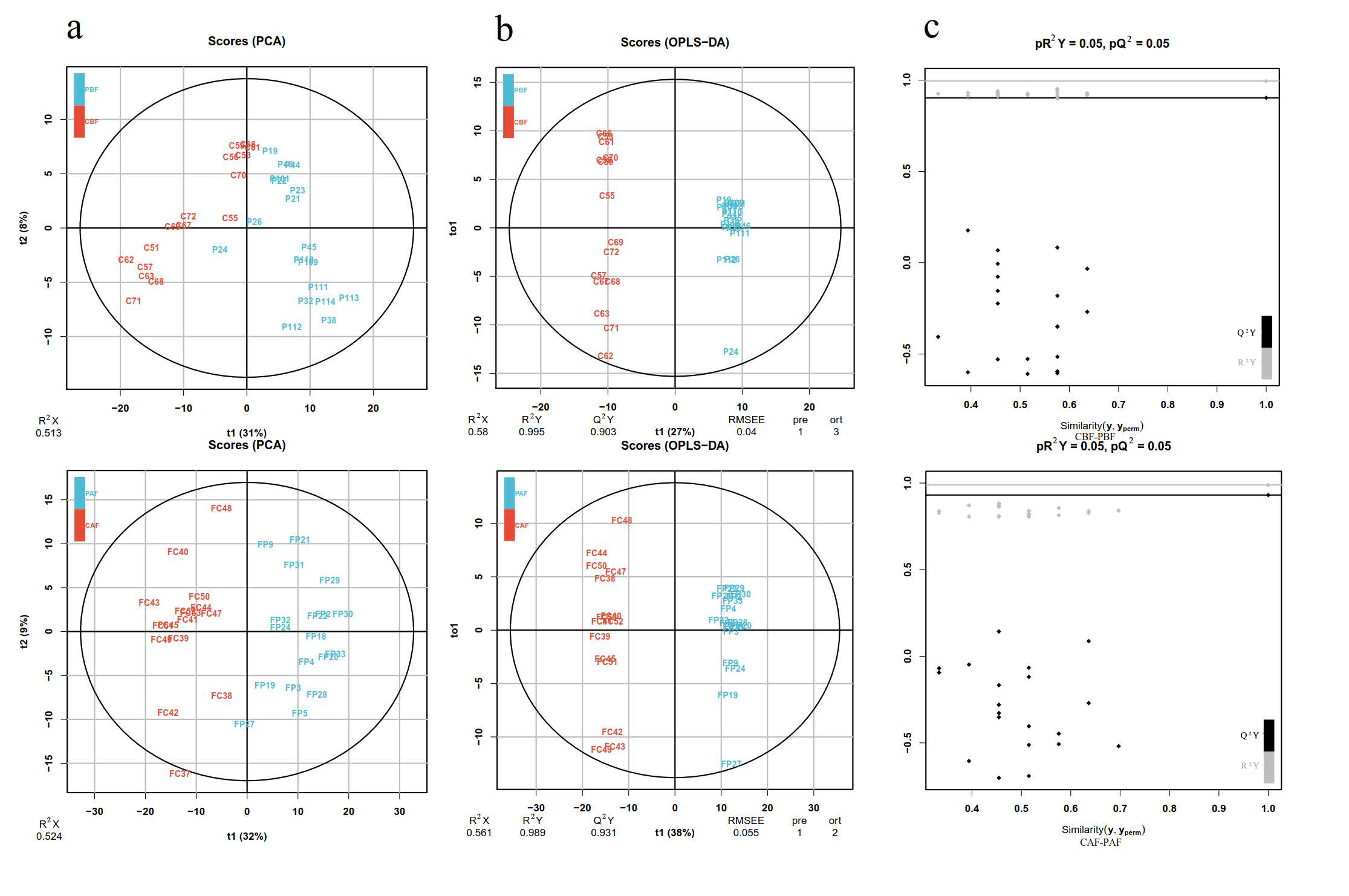

Supplement: Supplementary Figure 1 — (A) PCA analyses comparing metabolic profiles of subjects within Ethnic groups (CBF vs. PBF; CAF vs. PAF. CBF/PBF, Chinese or Pakistani before fasting; CAF/PAF, Chinese or Pakistani after fasting). Each numbered datapoint represents an individual subject. Blue, Pakistani; Red, Chinese. (B) OPLS-DA scores of fasting groups showed significant differences in clustering between ethnic groups (CBF vs. PBF; CAF vs. PAF). The abscissa represents the predicted principal component score of the first principal component and the ordinate represents the variability within the grouping. R2 represents the explanatory power of the model to variables, and Q2 represents the predictability of the model. (C) Model validation of OPLS-DA (CBF vs. PBF; CAF vs. PAF) (p < 0.05). The abscissa indicates similarity with the original model, and the ordinates represent R2Y and Q values. Gray and black points on the respective lines in the upper right corner indicate the actual values, while points on the left represent simulated values. When all simulated Q2 (black) and R2 values (gray) are lower than the respective original points, the model is robust, without over-fitting. [file Image_1.TIF]

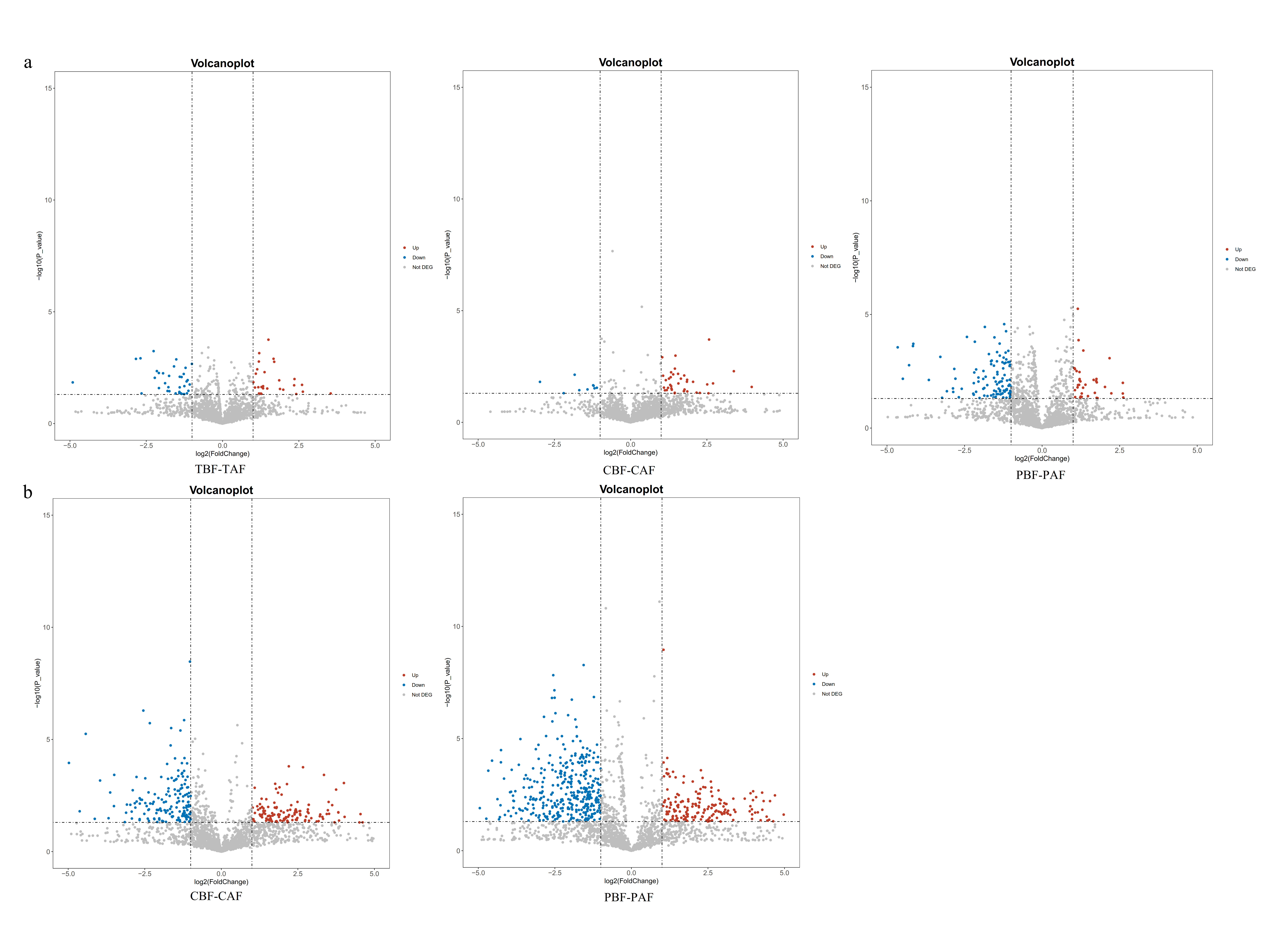

Supplement: Supplementary Figure 2 — (A) The volcano figure of fasting groups (TBF vs. TAF; CBF vs. CAF; PBF vs. PAF. CBF/CAF, Chinese before or after fasting; PBF/PAF, Pakistani before or after fasting; TBF/TAF, Total subjects before or after fasting) shows the number of different metabolites detected in different fasting groups (VIP > 1, p < 0.05), with red representing up-regulated metabolites and blue representing down-regulated metabolites. (B) The volcano figure of ethnic groups (CBF vs. PBF; CAF vs. PAF. CBF/PBF, Chinese or Pakistani before fasting; CAF/PAF, Chinese or Pakistani after fasting) shows the number of different metabolites detected in different ethnic groups (VIP > 1, p < 0.05), with red representing up-regulated metabolites and blue representing down-regulated metabolites. [file Image_2.TIF]

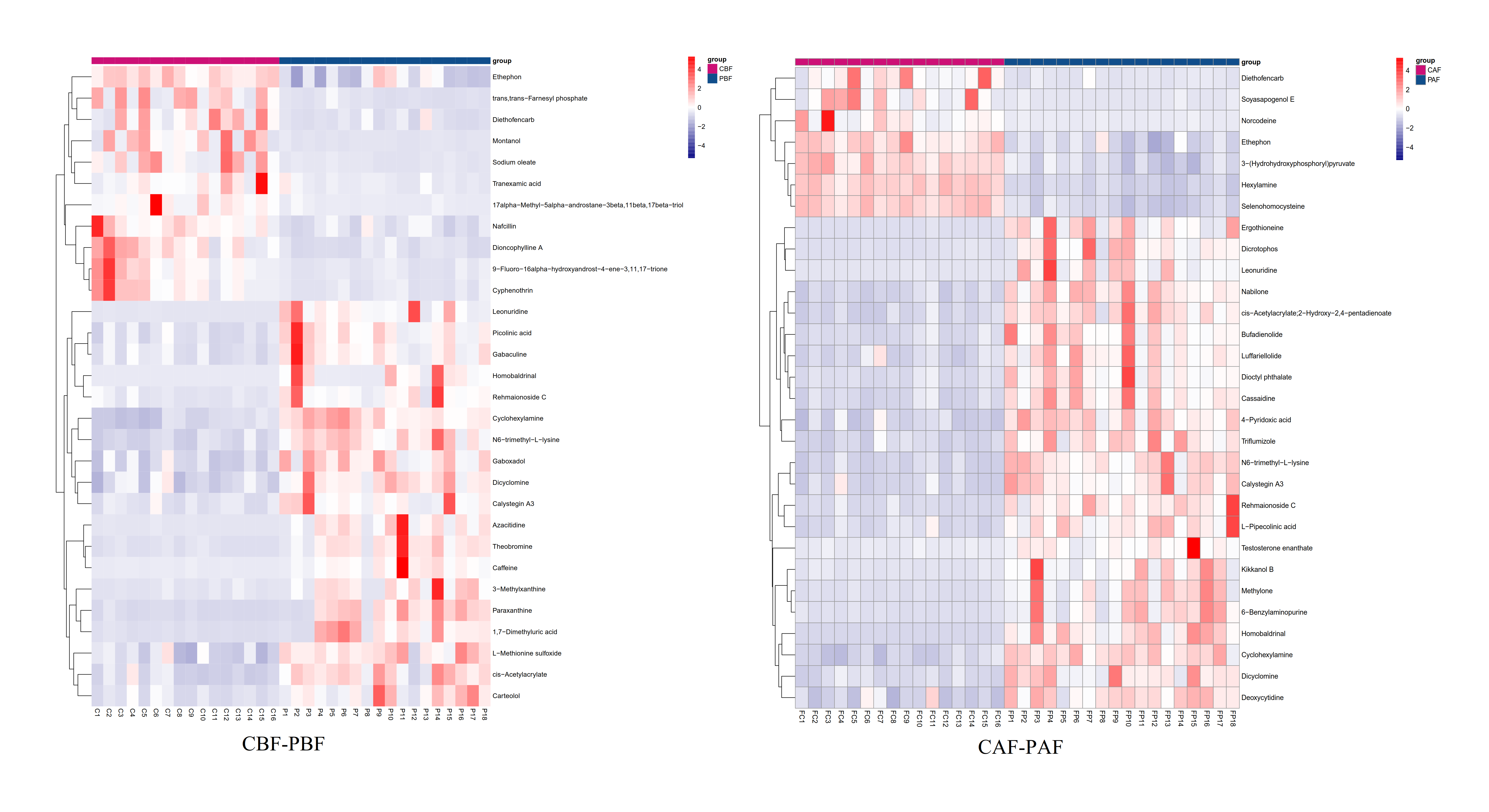

Supplement: Supplementary Figure 3 — Heat maps of metabolites of ethnic groups (CBF vs. PBF; CAF vs. PAF. CBF/PBF, Chinese or Pakistani before fasting; CAF/PAF, Chinese or Pakistani after fasting) show the first 30 metabolite that was significantly different between Chinese and the Pakistanis (p < 0.05). [file Image_3.TIF]

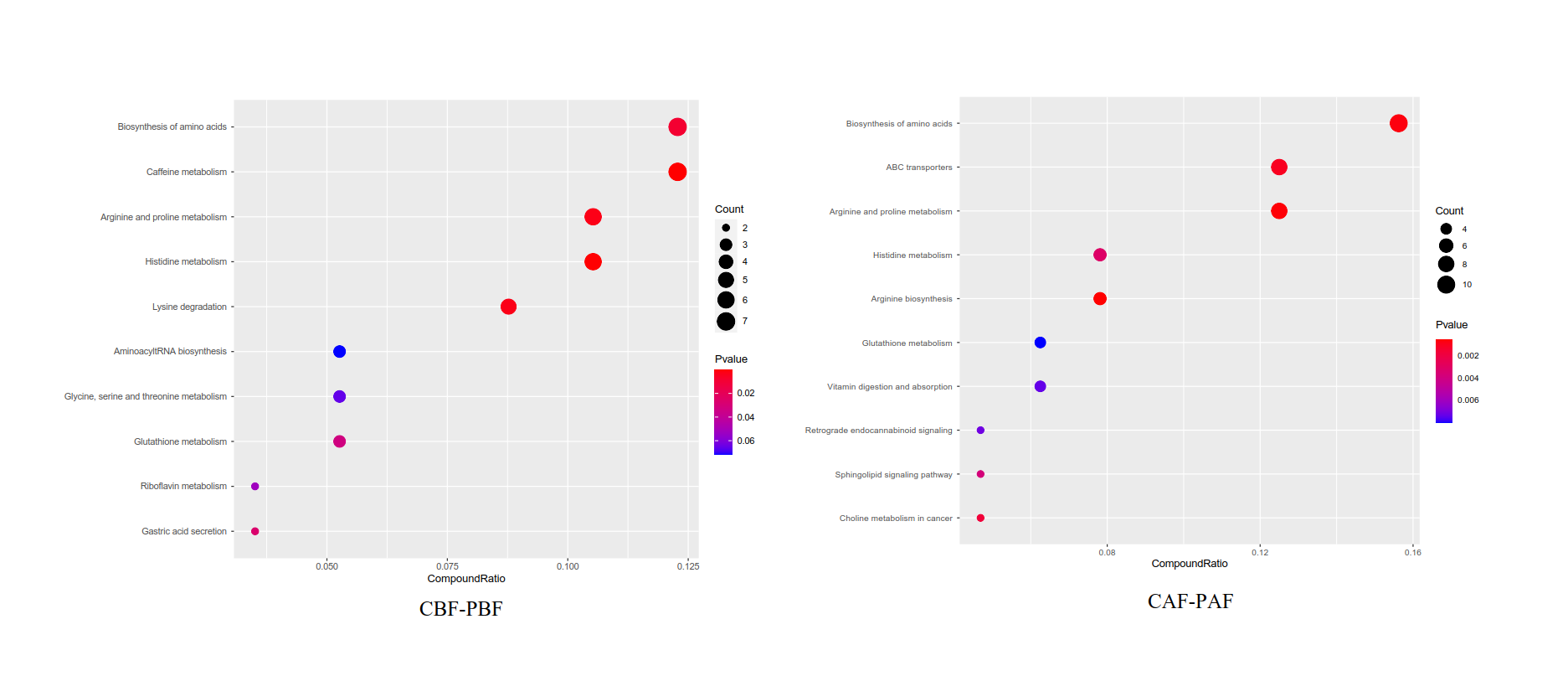

Supplement: Supplementary Figure 4 — Dot plot of top 10 metabolite pathway 619 of ethnic groups (CBF vs. PBF; CAF vs. PAF. CBF/PBF, Chinese or Pakistani before fasting; CAF/PAF, Chinese or Pakistani after fasting). The abscissa is the proportion of metabolites, and the ordinate is the path. The redder the color of the point, the smaller the p value, and the size of the point represents the quantity of metabolites. [file Image_4.TIF]

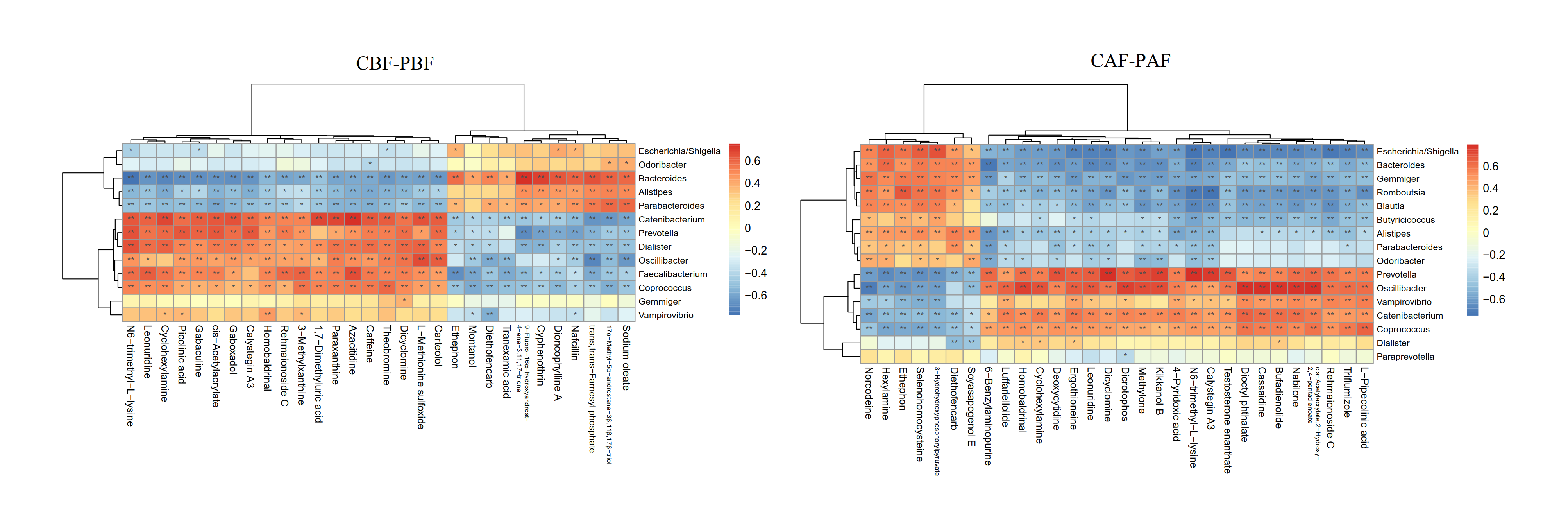

Supplement: Supplementary Figure 5 — Correlation between fecal microbiota members and metabolites between of ethnic groups (CBF vs. PBF; CAF vs. PAF. CBF/PBF, Chinese or Pakistani before fasting; CAF/PAF, Chinese or Pakistani after fasting). The abscissa represents the top 30 differential metabolites, and the ordinate represents the highest abundance species of intestinal microflora (genus level). Black stars in the box indicate significant results (*p < 0.05; **p < 0.01). [file Image_5.TIF]

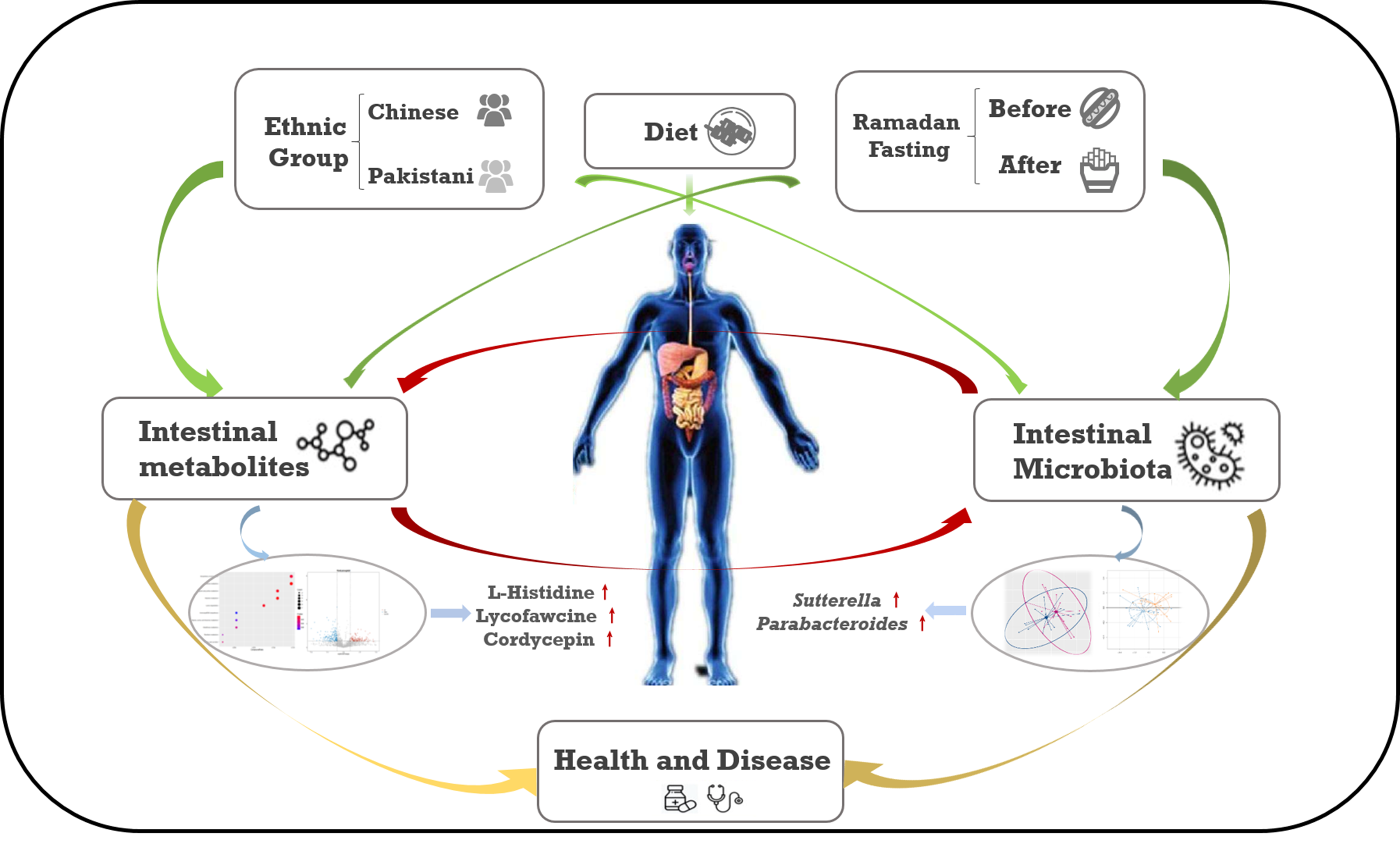

Supplement: Supplementary Figure 6 — Graphical Abstract: Bridging human health, gut microbiota & metabolites by fasting. [file Image_6.TIF]
