# Supplementary material for: Shifts in Fecal Metabolite Profiles Associated With Ramadan Fasting Among Chinese and Pakistani Individuals
Source: Front Nutr. 2022 May 3;9:845086. doi: 10.3389/fnut.2022.845086 (PMC9113920; doi:10.3389/fnut.2022.845086)
Supplement: Supplementary file 7 [file Table_1.DOCX]

Supplement Table 1. BMI of all the subjects before and after fasting in different groups

| BMI | N | (Mean ± SD) | *p* |
| --- | --- | --- | --- |
| TBF | 34 | 25.29 ± 3.80 | 0.85 |
| TAF | 34 | 21.49 ± 3.85 |  |
| CBF | 16 | 26.76 ± 0.78 | 0.07 |
| CAF | 16 | 20.26 ± 1.71 |  |
| PBF | 18 | 23.97 ± 4.86 | 0.94 |
| PAF | 18 | 22.59 ± 4.85 |  |
| CBF | 16 | 26.76 ± 0.78 | 0.01 |
| PBF | 18 | 23.97 ± 4.86 |  |
| CAF | 16 | 20.26 ± 1.71 | 0.07 |
| PAF | 18 | 22.59 ± 4.85 |  |

BMI: body mass index. TBF/TAF, Total subjects before or after fasting; CBF/CAF, Chinese before or after fasting; PBF/PAF, Pakistani before or after fasting; CBF/PBF, Chinese or Pakistani before fasting; CAF/PAF, Chinese or Pakistani after fasting. P value was calculated using Student’s t-test.
